# QEX: target-specific druglikeness filter enhances ligand-based virtual screening

**DOI:** 10.1007/s11030-018-9842-3

**Published:** 2018-07-03

**Authors:** Masahiro Mochizuki, Shogo D. Suzuki, Keisuke Yanagisawa, Masahito Ohue, Yutaka Akiyama

**Affiliations:** 1 IMSBIO, Co., Ltd, Owl tower 6F, 4-21-1, Higashi-ikebukuro, Toshima-ku, Tokyo, 170-0013 Japan; 2 School of Computing, Tokyo Institute of Technology, 2-12-1 W8-76, Ookayama, Meguro-ku, Tokyo, 152-8550 Japan; 3 Educational Academy of Computational Life Sciences (ACLS), Tokyo Institute of Technology, 2-12-1 W8-93, Ookayama, Meguro-ku, Tokyo, 152-8550 Japan; 4 Advanced Computational Drug Discovery Unit (ACDD), Institute of Innovative Research, Tokyo Institute of Technology, 4259 Nagatutacho, Midori-ku, Yokohama, Kanagawa 226-8501 Japan

**Keywords:** Computational drug discovery, Druglikeness, Virtual screening, Quantitative estimate of druglikeness (QED), QEX

## Abstract

Druglikeness is a useful concept for screening drug candidate compounds. We developed QEX, which is a new druglikeness index specific to individual targets. QEX is an improvement of the quantitative estimate of druglikeness (QED) method, which is a popular quantitative evaluation method of druglikeness proposed by Bickerton et al. QEX models the physicochemical properties of compounds that act on each target protein based on the concept of QED modeling physicochemical properties from information on US Food and Drug Administration-approved drugs. The result of the evaluation of PubChem assay data revealed that QEX showed better performance than the original QED did (the area under the curve value of the receiver operating characteristic curve improved by 0.069-0.236). We also present the c-Src inhibitor filtering results of the QEX constructed using Src family kinase inhibitors as a case study. QEX distinguished the inhibitors and non-inhibitors better than QED did. QEX works efficiently even when datasets of inactive compounds are unavailable. If both active and inactive compounds are present, QEX can be used as an initial filter to enhance the screening ability of conventional ligand-based virtual screenings.

**Electronic supplementary material:**

The online version of this article (10.1007/s11030-018-9842-3) contains supplementary material, which is available to authorized users.

## Introduction

Drug molecules are known to share similar physicochemical properties. Molecules possessing these properties are called *druglike*. Druglikeness is a useful and simple criterion to screen potential drug molecules. The most popular method for evaluating druglikeness is Lipinski’s rule (rule of five, RO5) [1], a rule of thumb focusing on orally administered drugs. The rule consists of criteria related to the following four properties:

- The number of hydrogen bond acceptors (HBA) is no more than 5.
- The number of hydrogen bond donors (HBD) is no more than 5.
- The molecular weight (MW) is less than 500.
- The calculated value of the logarithm of the octanol–water partition coefficient (CLogP) is less than 5.

Molecules that fulfill all the criteria are determined to possess druglikeness. The outcome of Lipinski’s rule is basically binary, i.e., whether druglike or not, while the number of fulfilled criteria can be used as a multistage evaluation of druglikeness. In contrast, the quantitative estimate of druglikeness (QED) [2] proposed by Bickerton et al. [3] provides continuous scores of druglikeness. QED is based on eight properties: HBA, HBD, MW (which also appear in RO5), LogP value estimated using the Ghose–Crippen method [3] (ALogP), molecular polar surface area (PSA), as well as numbers of rotatable bonds (ROTB), aromatic rings (AROM), and structural alerts [4] (ALERTS). A QED score is calculated using the geometric mean of desirability functions [5], each of which corresponds to an individual property. The functions are modeled as asymmetric sigmoidal functions and fitted to the histogram of a corresponding physicochemical property of the oral drug. Since each function is adjusted to a maximum value of 1, the QED score is also between 0 and 1. Consequently, a higher QED score indicates the compounds are more favorable as a drug.

After QED, Yusof and Segal [6] developed another quantitative estimation method called relative drug likelihood (RDL). While the QED method is based on similarity to known drugs, RDL focuses on differences between drug and non-drug compounds. The RDL score is calculated as the geometric mean of relative likelihoods instead of the simple desirability functions. Therefore, the relative likelihood is a ratio of the posterior probability of a compound being a drug to that of not being one, which is derived from Bayes’ theorem.

The QED approach assesses the similarity of a substance to known US Food and Drug Administration (FDA)-approved drugs, i.e., 771 drugs curated by its authors, which constitute a heterogeneous mixture of drugs. Favorable properties for drugs depend on the characteristics of the target protein. For instance, the MW of a drug is thought to be affected not only by the constraint to maintain permeability but also by the volume of the binding pocket. Indeed, distributions of QED scores of drugs vary depending on their targets [2], suggesting that QED is not an optimal method for every target and there is room for improvement of at least some targets. Therefore, we proposed a target-specific QED, named QEX, which specifically screens drug candidates directed at particular targets. Although RDL has been shown to apply to specific objectives such as screening of orally administrated G protein-coupled receptor (GPCR) inhibitors, a dataset of inactive compounds required by modeling of RDL is not necessarily available in other cases. In contrast, since QEX and the original QED can be modeled with only the active compound, it can be used even in cases lacking inactive compounds. In this study, the effectiveness of QEX was examined using several targets, in comparison with the original QED.

## Results and discussion

### QEX outperforms the original QED for individual targets

The screening ability of QEX was examined by cross-validating five targets. The benchmark screening scores of QEX, the original QED, and Lipinski’s RO5 are shown in Table 1a, b, and Table S1 in Supplementary Material 1, respectively. The QEX performed better than the original QED and RO5 did in every case shown in Table 1 and Table S1. As shown in Table S2 and Figure S1, RO5 passed most of the compounds, thus showing poor screening ability. The result indicates that QEX has an advantage in being able to screen active compounds for a specific target.

**Table 1:** Comparison of screening scores between QEX and the original quantitative estimate of druglikeness (QED). Benchmark results of screening using (a) QEX models specialized for each of five targets and (b) original QED model

(a) QEX (proposed)							
Target	AUC	EF (1%)	EF (2%)	EF (5%)	EF (10%)	EF (20%)	EF (50%)
Streptokinase	0.678	2.387	2.365	2.477	2.230	1.991	1.489
PP1	0.668	3.473	3.126	2.582	2.462	2.085	1.450
TIM10	0.744	3.196	2.975	2.754	2.533	2.302	1.696
SENP8	0.777	5.580	5.038	4.335	3.557	2.722	1.743
KCNK9	0.700	2.527	2.503	2.623	2.313	2.008	1.566

(b) Original QED							
Target	AUC	EF (1%)	EF (2%)	EF (5%)	EF (10%)	EF (20%)	EF (50%)
Streptokinase	0.485	0.586	0.563	0.586	0.545	0.635	0.922
PP1	0.432	0.299	0.149	0.358	0.397	0.541	0.757
TIM10	0.599	0.952	0.850	0.959	0.976	1.080	1.279
SENP8	0.708	3.251	3.211	2.770	2.124	1.640	1.631
KCNK9	0.497	0.381	0.381	0.610	0.649	0.722	0.957

PP1, protein phosphatase 1; TIM10, translocate of the inner mitochondrial membrane subunit 10; SENP8, sentrin-specific protease 8; KCNK9, potassium two-pore domain channel subfamily K member 9; AUC, area under the curve; EF, enrichment factor

The properties that showed peaks of distribution are shown in Table 2. Theoretically, they indicate the ideal values of the ability of each physicochemical property to inhibit a corresponding target, because compounds possessing that property are the most frequent in datasets of known inhibitors. Thus, these properties are assumed to reflect the nature of the target protein, especially the inhibitor-binding pocket. In addition, peak values of the original QED but not QEX are also supposed to reflect absorption, distribution, metabolism, excretion, and toxicity (ADMET) because the original QED is trained with FDA-approved oral drugs. For instance, the peak value of LogP of the original QED model is lower than that of any QEX model in Table 2, suggesting that low lipophilicity and high hydrophilicity are important for orally absorbed drugs. Then, it can be assumed that the original QED and QEX have different roles in the process of drug discovery.

**Table 2:** Distribution peaks of each physicochemical property. Properties showing peaks of curve fitted to its distribution are shown for each QEX model and the original quantitative estimate of druglikeness (QED)

Target	MW	ALogP	HBD	HBA	PSA	ROTB	AROM	ALERTS
Streptokinase	367.0	4.27	0.62	4.78	71.5	3.73	2.8	− 4.5
PP1	383.6	4.10	0.75	5.44	79.8	4.04	3.2	− 236.2
TIM10	315.0	3.61	1.11	4.10	57.8	3.26	2.1	− 24.6
SENP8	269.0	3.41	1.14	3.75	54.1	2.47	2.0	− 117.9
KCNK9	375.6	4.53	0.90	4.40	54.8	4.93	2.9	− 144.2
Original QED	305.0	2.70	1.19	2.38	57.3	3.03	1.8	− 24.6

PP1, protein phosphatase 1; TIM10, translocate of the inner mitochondrial membrane subunit 10; SENP8, sentrin-specific protease 8; KCNK9, potassium two-pore domain channel subfamily K member 9; MW, molecular weight; ALogP, LogP value estimated using Ghose–Crippen method; HBD, hydrogen bond donors; HBA, hydrogen bond acceptors; PSA, polar surface area; ROTB, rotatable bonds; AROM, aromatic rings; ALERTS, structural alerts; QED, quantitative estimate of druglikeness

An advantage of QEX is that its model is only trained with a dataset of active compounds. In other words, QEX does not require a dataset of inactive compounds, which are often difficult to obtain in large numbers from public databases [7, 8]. If the examples of inactive compounds provided are insufficient, the performance of the machine learning classifier would be worse. In that situation, QEX could be more effective than the machine learning method is. Compared to the original QED oriented to oral drugs, QEX is suitable for screening lead compounds acting on a specific target.

### Application to c-Src inhibitor screening

To further assess the QEX, we used it to screen for c-Src inhibitors. c-Src is a tyrosine kinase, and many cellular processes are driven by the activation and inactivation of protein tyrosine kinases through phosphorylation. The interplay of c-Src and other proteins has been widely studied, and its role in pluripotent embryonic stem cells has also been reported [9]. Thus, inhibitors of c-Src have been identified using computational and experimental techniques [10].

For the compound screening, a known inhibitor library was obtained from Chiba et al. [11], and a QEX model for c-Src was built using these inhibitors. Then, we applied our QEX and the QED models to three popular c-Src inhibitors, PP2, gefitinib, and sunitinib, and three non-inhibitors, oseltamivir, aspirin, and arginine. The resulting QEX and QED scores (Table 3) show that the QEX model distinguished the inhibitors and non-inhibitors better than the QED model did. In particular, the QEX model showed a low score not only for arginine with its low druglikeness, but also for oseltamivir and aspirin, which are not c-Src inhibitors.

**Table 3:**
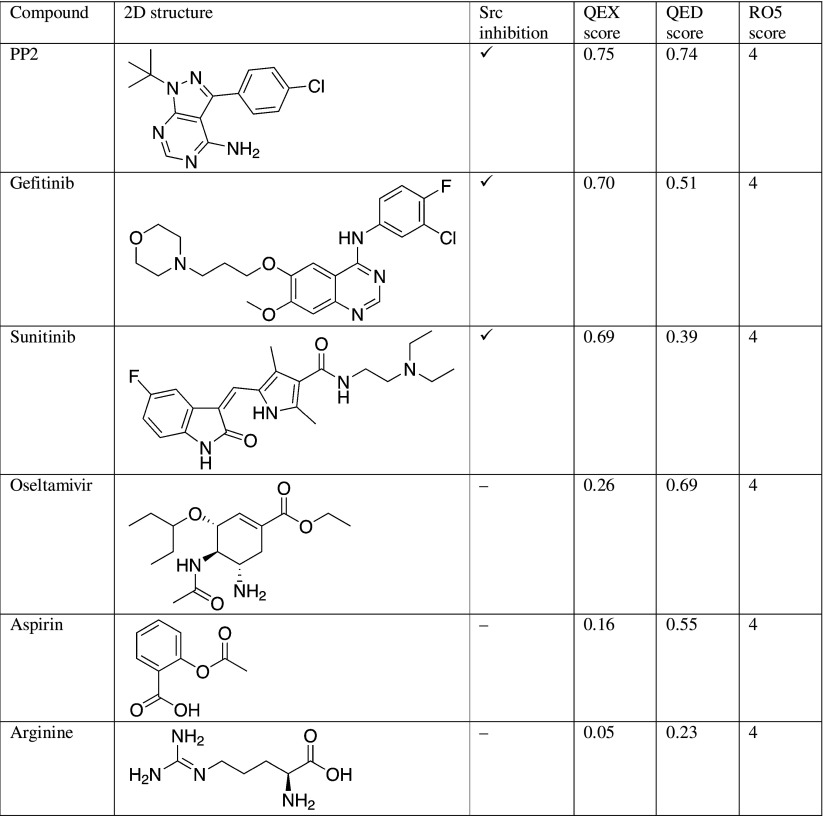
QEX, quantitative estimates of druglikeness (QED), and Lipinski’s rule of five (RO5) scores for c-Src inhibitors and non-inhibitors

## Conclusions

QEX was better suited for screening inhibitors of specific targets than the original QED. QEX is easy to use when datasets of inactive compounds are not available. If both active and inactive compounds are available, QEX can be used as an initial filter to enhance conventional ligand-based virtual screenings.

The concept of QEX could be expanded beyond determination of druglikeness. Indeed, Limmer and Burken [12] developed desirability functions to describe chemical transport across plant root–soil boundaries based on the concept of QED, which are called the quantitative estimates of plant translocation (QEPT) [12]. Thus, it is possible to target-specific QEPT using numerous data entries, which could contribute to phytoremediation efforts and herbicide design. Finally, this topic is one of our proposed future studies.

## Materials and methods

### Calculation of QEX and original QED values

A QEX was calculated using a procedure that was basically identical to that of the original QED, except that the QEX was modeled using compounds targeting a particular protein or protein family. The algorithm used is briefly described below. In the initial modeling steps, each of the eight physicochemical properties (MW, ALogP, HBD, HBA, PSA, ROTB, AROM, and ALERTS) was computed from a dataset of known active compounds using RDKit [13] version 2015.03.1. Then, a histogram of each property was constructed and was fitted to the asymmetric double sigmoidal function *Q*(*x*) shown in Eq. (1) by implementing the Levenberg–Marquardt algorithm in SciPy [14].

1 $$ Q(x) = a + \frac{b}{{1 + \exp \left( { - \tfrac{{x - c + \tfrac{d}{2}}}{e}} \right)}}\left[ {1 - \frac{1}{{1 + \exp \left( { - \tfrac{{x - c - \tfrac{d}{2}}}{f}} \right)}}} \right] $$

All the fitted functions ($$ Q_{\text{MW}} (x),Q_{\text{ALogP}} (x), \ldots $$) were divided by the maximum values so as to adjust the maximum divided function to 1. The divided function $$ \tilde{Q}_{i} (x) $$$$ (i \in \mathcal{\mathcal{C}},\,\mathcal{\mathcal{C}} = \{ {\text{MW}},\,{\text{ALogP}},\,{\text{HBD}},\,{\text{HBA}},\,{\text{ROTB}},\,{\text{AROM}},\,{\text{ALERTS}}\} ) $$ was used as the desirability function, and a QEX score was assigned as the weighted geometric mean of all desirability functions as shown in Eq. (2).

2 $$ {\text{QEX}}\,{\text{score}} = \exp \left( {\frac{{\sum\nolimits_{{i \in \mathcal{\mathcal{C}}}} {w_{i} \ln (\tilde{Q}_{i} )} }}{{\sum\nolimits_{{i \in \mathcal{\mathcal{C}}}} {w_{i} } }}} \right) $$

All the eight weights were exhaustively tried from 0 to 1 in increments of 0.25, and the mean of the 1000 weight combinations which provided the highest Shannon entropy was adopted here. A Shannon entropy of a model was calculated as shown in Eq. (3).

3 $$ {\text{Entropy}} = - \sum\limits_{k = 1}^{n} {({\text{QEX}}\,{\text{score}}_{k} )\log_{2} ({\text{QEX}}\,{\text{score}}_{k} )} $$

where *n* is the number of compounds used for modeling.

The original QED values in this study were also calculated using the same implementation used for the QEX but were modeled using 771 FDA-approved drugs curated by Bickerton et al. [2] (Supplementary Material 2).

### Dataset

All assayed compound data for the five target proteins were obtained from PubChem [15]. Table 4 shows each target as well as the numbers of active (positive) and inactive (negative) compounds. All compound structure data can be downloaded in SDF (structure data file) format in Supplementary material 3, 5, 7, 9, and 11. Their label information is in Supplementary material 4, 6, 8, 10, and 12. Building the QEX model only requires active compounds while inactive compounds were used only for evaluating the prediction performance of RO5, QED, and QEX.

**Table 4:** Dataset for evaluation of QEX performances. All compound data are available in Supplementary Materials

PubChem AID	Target name	Active (#)	Inactive (#)
1915	Streptokinase	2220	1017
2358	PP1	1007	937
463215	TIM10	2941	1695
488912	SENP8	2491	3705
492992	KCKN9	2097	2820

PP1, protein phosphatase 1; TIM10, translocate of the inner mitochondrial membrane subunit 10; SENP8, sentrin-specific protease 8; KCNK9, potassium two-pore domain channel subfamily K member 9

### Validation of druglikeness screening performance

The AUC of the ROC [16] and early EF [17] are considered in evaluating screening performances, which are generally used in virtual screening studies. In this study, we used a list of experimentally verified active and inactive compounds (positive and negative samples, respectively). These positives and negatives were further categorized as true or false according to their rank above or below a certain threshold of the QEX and QED filtering results. Therefore, the actives ranked above a chosen threshold were considered true positives. In contrast, RO5 rankings are based on the number of rules passed. To generate the ROC curve, the true positive ratio (TPR = TP/(TP + FN)) and false positive ratio (FPR = FP/(TN + FP)) were calculated, where TP, FP, FN, and TN are the number of true positives, false positives, false negatives, and true negatives, respectively. In the ROC curve, the TPR was plotted as a function of the FPR. The AUC was then calculated to assess the quantitative performance of different QEX and QED models. An AUC of 0.5 corresponded to a random selection of the compounds using the target.

The EF (*x*%) value indicates how much more often an active compound is ranked in the top *x*% of a screening result than it is randomly selected, i.e., the times the dataset is enriched. Specifically, the EF was calculated using Eq. (4):

4 $$ {\text{EF}}\,(x\% ) = \frac{{n_{ \exp }^{x\% } }}{N \times x\% } $$

where *n*_exp_^*x*%^ is the number of experimentally verified actives in the top *x*% of the database and *N* is the total number of actives in the database. In this study, EF (1%), EF (2%), EF (5%), EF (10%), EF (20%), and EF (50%) were calculated from the top 1, 2, 5, 10, 20, and 50% of the screening results, respectively.

**Fig. 1:**
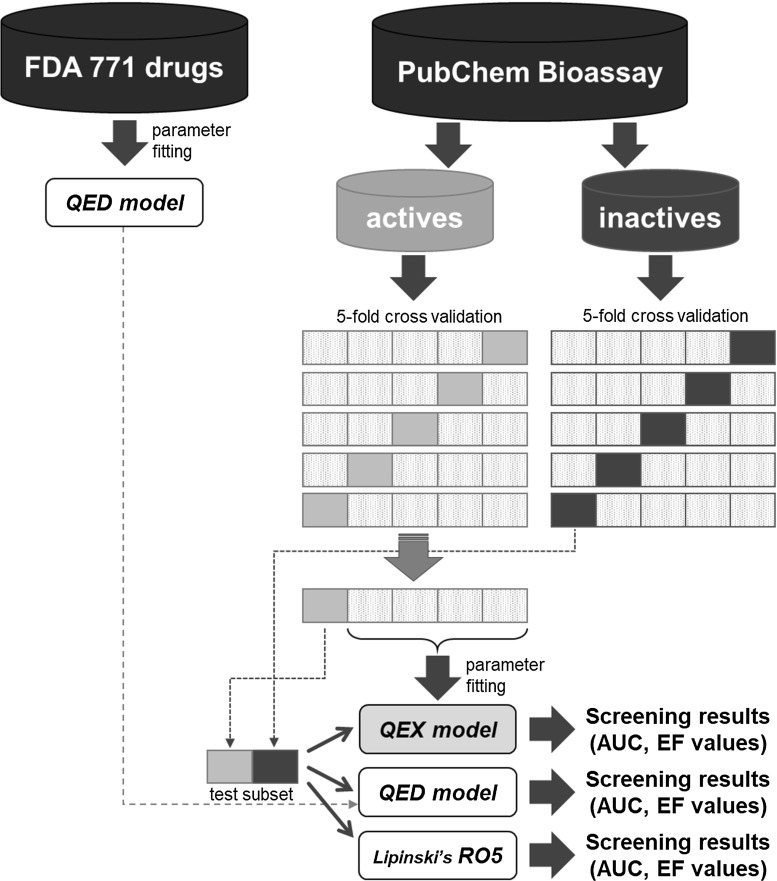
Overview of dataset construction and cross-validation for evaluating Lipinski’s rule of five (RO5), quantitative estimate of druglikeness (QED), and QEX models. FDA, US Food and Drug Administration; AUC, area under the curve; EF, enrichment factor

Learning and evaluation of the QEX model function were performed using 5-fold cross-validation. Specifically, the active compounds were divided into five subsets, and the parameters of the fitting functions were determined using four of the five subsets, and the AUC and EF of the remaining subset were obtained. In addition, the QED model, which was constructed in advance using 771 FDA-approved drugs, was also applied to the same subset. The AUC and EF values shown in Table 1 were the average of five validations obtained from five subsets. An overview of the dataset and the validation method is shown in Fig. 1.

### Application to c-Src inhibitor screening

Experimentally determined inhibitors of Src family kinases were obtained to construct a Src-specific QEX model for major c-Src inhibitors and irrelevant compounds, which was then compared with the QED model. Inhibitors of Src family kinases were published by Chiba et al. [11, 18] through the second computer-aided drug discovery contest of the Initiative for Parallel Bioinformatics (IPAB) [19]. The target Src family consists of ten proteins shown in Table 5. They were extracted using ChEMBL version 19 [21] and BindingDB [22]. The extraction criteria were as follows: half-maximal inhibitory concentration (IC_50_) < 10 μmol L^−1^, *K*_*i*_ < 10 μmol L^−1^, *K*_*d*_ < 10 μmol L^−1^, and inhibition rates > 30%, whereas the experimental conditions were not considered. Finally, 3528 unique compounds were identified. They are available in Supplementary material 13 (Src_inhibitors.sdf) and can be obtained from the IPAB Web site [19].

**Table 5:** Src family proteins obtained from ChEMBL [20]

ChEMBL ID	Target molecule
CHEMBL4223	Tyrosine-protein kinase FRK
CHEMBL3234	Tyrosine-protein kinase HCK
CHEMBL3905	Tyrosine-protein kinase LYN
CHEMBL2250	Tyrosine-protein kinase BLK
CHEMBL258	Tyrosine-protein kinase LCK
CHEMBL4454	Tyrosine-protein kinase FGR
CHEMBL5703	Tyrosine-protein kinase SRMS
CHEMBL1841	Tyrosine-protein kinase FYN
CHEMBL267	Tyrosine-protein kinase SRC
CHEMBL2073	Tyrosine-protein kinase YES

## Electronic supplementary material

Below is the link to the electronic supplementary material.

Supplementary material 1 (PDF 353 kb)

Supplementary material 2 (SDF 1855 kb)

Supplementary material 3 (SDF 10560 kb)

Supplementary material 4 (CSV 300 kb)

Supplementary material 5 (SDF 6836 kb)

Supplementary material 6 (CSV 92 kb)

Supplementary material 7 (SDF 16132 kb)

Supplementary material 8 (CSV 280 kb)

Supplementary material 9 (SDF 17032 kb)

Supplementary material 10 (CSV 692 kb)

Supplementary material 11 (SDF 13809 kb)

Supplementary material 12 (CSV 387 kb)

Supplementary material 13 (SDF 7218 kb)

## Abbreviations

QED: Quantitative estimate of druglikeness
RO5: Lipinski’s rule of five
HBA: Number of hydrogen bond acceptors
HBD: Number of hydrogen bond donors
MW: Molecular weight
ALogP: LogP value estimated by Ghose–Crippen method
PSA: Molecular polar surface area
ROTB: Number of rotatable bonds
AROM: Number of aromatic rings
ALERTS: Number of structural alerts
RDL: Relative drug likelihood
FDA: US Food and Drug Administration
AUC: Area under the curve
ROC: Receiver operation characteristics
EF: Enrichment factor
QEPT: Quantitative estimate of plant translocation
TP: True positive
FP: False positive
FN: False negative
TN: True negative
TPR: True positive rate
FPR: False positive rate
