# Supplementary material for: QEX: target-specific druglikeness filter enhances ligand-based virtual screening
Source: Mol Divers. 2018 Jul 3;23(1):11–8. doi: 10.1007/s11030-018-9842-3 (PMC6394530; doi:10.1007/s11030-018-9842-3)
Supplement: Supplementary file 1 — Supplementary material 1 (PDF 353 kb) [file 11030_2018_9842_MOESM1_ESM.pdf]

Supplementary Materials

QEX: Target-specific druglikeness filter enhances ligand-based virtual screening

Masahiro Mochizuki[1,3], Shogo D. Suzuki[2,3], Keisuke Yanagisawa[2,3], Masahito Ohue[2,4] and Yutaka Akiyama[2,3,4,*]

1 IMSBIO, Co., Ltd. Owl tower 6F, 4-21-1, Higashi-ikebukuro, Toshima-ku, Tokyo 170-0013, Japan.

2 School of Computing, Tokyo Institute of Technology. 2-12-1 W8-76, Ookayama, Meguro-ku, Tokyo 152-8550, Japan.

3 Educational Academy of Computational Life Sciences (ACLS), Tokyo Institute of Technology. 2-12-1 W8-93, Ookayama, Meguro-ku, Tokyo 152-8550, Japan.

4 Advanced Computational Drug Discovery Unit (ACDD), Institute of Innovative Research, Tokyo Institute of Technology. 4259 Nagatutacho, Midori-ku, Yokohama, Kanagawa 226-8501, Japan.

* Correspondence: akiyama@c.titech.ac.jp; Tel.: +81-3-5734-3645

Table S1. Screening scores of Lipinski's rule of five (RO5). The screening scores of RO5 were calculated based on the ranking arranged by the number of rules passed.

target	*AUC*	*EF (1%)*	*EF (2%)*	*EF (5%)*	*EF (10%)*	*EF (20%)*	*EF (50%)*
Streptokinase	0.463	0.970	0.970	0.970	0.970	0.970	0.970
PP1	0.497	0.994	0.994	0.994	0.994	0.994	0.994
TIM10	0.509	1.007	1.007	1.007	1.007	1.007	1.007
SENP8	0.516	1.020	1.020	1.020	1.020	1.020	1.020
KCNK9	0.445	0.916	0.916	0.916	0.916	0.916	0.916

PP1, protein phosphatase 1; TIM10, translocate of the inner mitochondrial membrane subunit 10; SENP8, sentrin-specific protease 8; KCNK9, potassium two-pore domain channel subfamily K member 9; AUC, area under the curve; EF, enrichment factor.

Note: The same values were found for all of EF percentages because more than half of the compounds passed all four RO5 rules in all targets, as shown in Table S2.

Table S2. Cross table of the number of compounds which passed and failed Lipinski's rule of five (RO5).

a. Streptokinase

	RO5 pass	RO5 fail
active	1,641	579
inactive	847	199

b. PPI

	RO5 pass	RO5 fail
active	735	272
inactive	692	245

c. TIM10

	RO5 pass	RO5 fail
active	2,658	283
inactive	1,502	193

d. SENP8

	RO5 pass	RO5 fail
active	2,449	42
inactive	3,523	182

e. KCNK9

	RO5 pass	RO5 fail
active	1,478	619
inactive	2,306	514

Figure S1. The distributions of the number of rules passed using Lipinski's rule of five (RO5).

a. Streptokinase

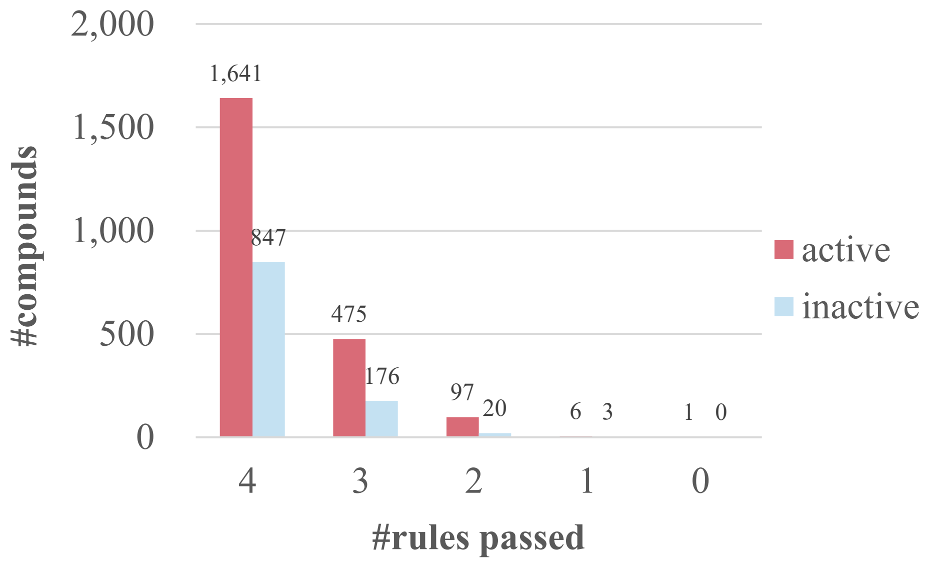

b. PPI

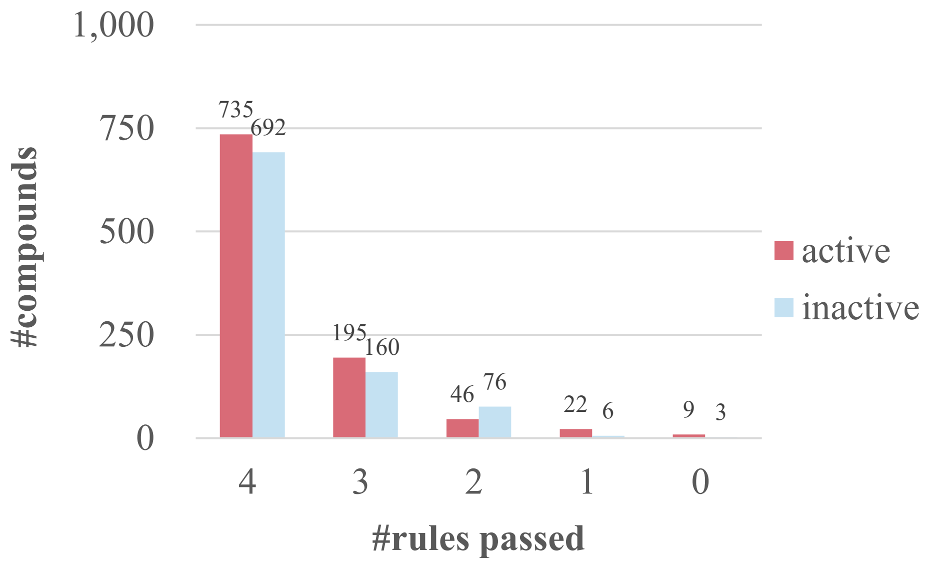

c. TIM10

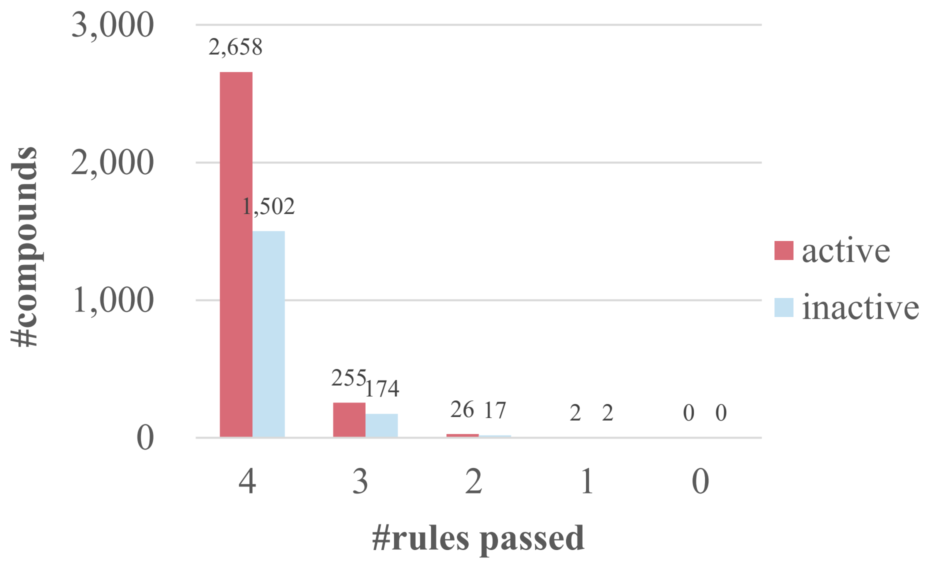

d. SENP8

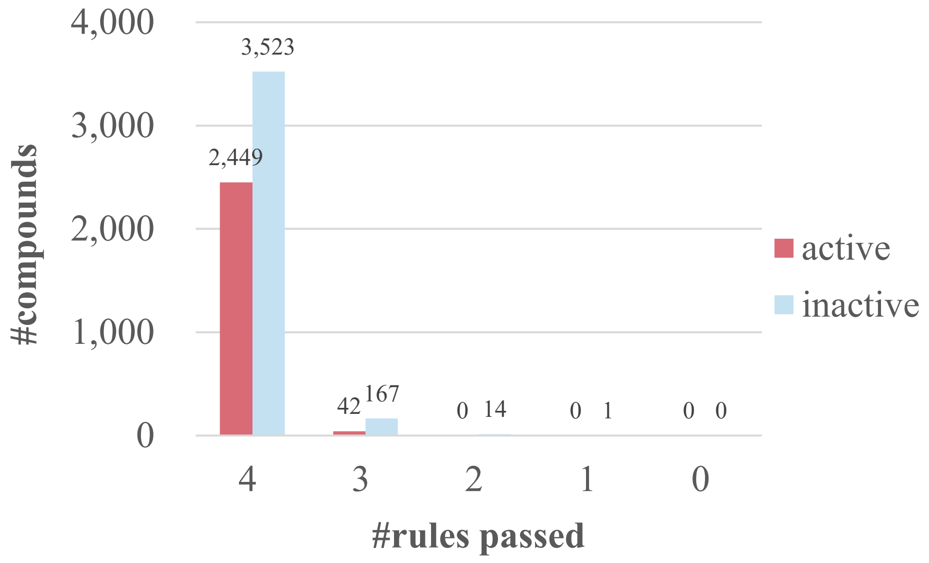

e. KCNK9

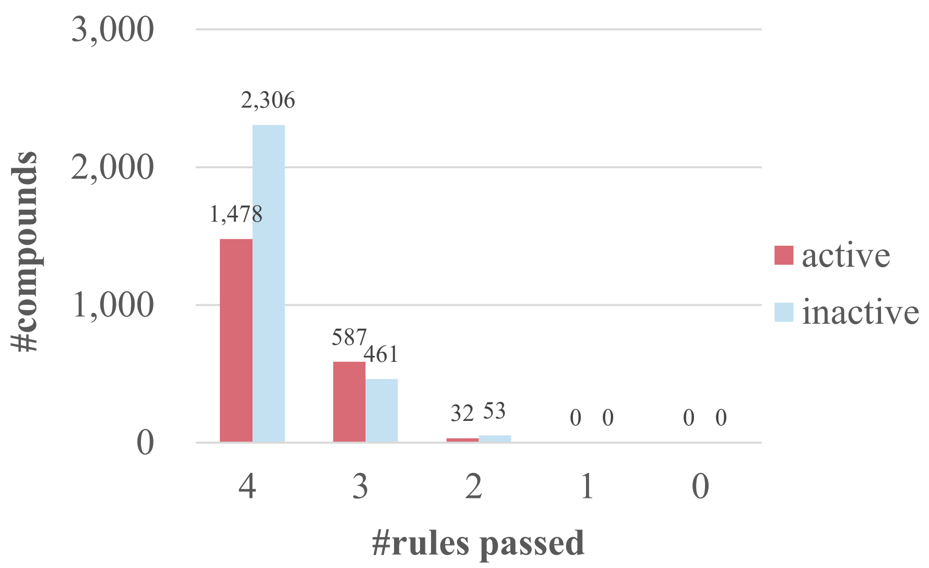

3,000
2,000
1,000
0
#compounds
2,306
1,478
587
461
32
53
0
0
0
0
4
3
2
1
0
#rules passed
active
inactive
